# Effects on Inter-Personal Memory of Dancing in Time with Others

**DOI:** 10.3389/fpsyg.2016.00167

**Published:** 2016-02-23

**Authors:** Matthew H. Woolhouse, Dan Tidhar, Ian Cross

**Affiliations:** ^1^Digital Music Lab, School of the Arts, McMaster University, HamiltonON, Canada; ^2^McMaster Institute for Music and the Mind, McMaster University, HamiltonON, Canada; ^3^Faculty of Music, University of CambridgeCambridge, UK

**Keywords:** music and dance, interpersonal entrainment, person perception, social bonding, silent disco, memory

## Abstract

We report an experiment investigating whether dancing to the same music enhances recall of person-related memory targets. The experiment used 40 dancers (all of whom were unaware of the experiment’s aim), two-channel silent-disco radio headphones, a marked-up dance floor, two types of music, and memory targets (sash colors and symbols). In each trial, 10 dancers wore radio headphones and one of four different colored sashes, half of which carried cat symbols. Using silent-disco technology, one type of music was surreptitiously transmitted to half the dancers, while music at a different tempo was transmitted to the remaining dancers. Pre-experiment, the dancers’ faces were photographed. Post-experiment, each dancer was presented with the photographs of the other dancers and asked to recall their memory targets. Results showed that same-music dancing significantly enhanced memory for sash color and sash symbol. Our findings are discussed in light of recent eye-movement research that showed significantly increased gaze durations for people observing music-dance synchrony versus music-dance asynchrony, and in relation to current literature on interpersonal entrainment, group cohesion, and social bonding.

## Introduction

Spontaneous coupling of behavior or coordinated joint-action in the absence of explicit instruction is an important feature of social interaction (Richardson and Dale, 2005; Sebanz et al., 2006). Repetitive and rhythmic movement synchrony between individuals, sometimes referred to as “interpersonal entrainment” (Clayton et al., 2005; Phillips-Silver and Keller, 2012), has been shown to have positive effects on perceived social relationships (Hove and Risen, 2009; Miles et al., 2009; Kirschner and Tomasello, 2010; Cirelli et al., 2014), and has been identified as an important factor in everyday social interaction (Shockley et al., 2003, 2009). In a series of studies, Wiltermuth and Heath (2009) showed that acting in synchrony with others increases cooperation by strengthening social attachment among group members. The positive effects on perceived social relationships found in research on person-to-person coordination fit well with accounts of group dancing in the ethnographic and sociological literature (Cross, 2008). In short, dancing is an important way to create situations in which people move either synchronously or in “sympathy”, thereby establishing and reinforce social bonds.

The positive social effects referred to above are also consistent with cognitive and evolutionary hypotheses concerning entrainment in dance and music^1^, which suggest that interpersonal entrainment helps people to direct their attentional foci toward one other (Large and Jones, 1999). In turn, coordinated, joint attention may support functions such as social bonding (Freeman, 2000; Merker, 2000; Brown et al., 2004; Cross, 2011), courtship (Catchpole and Slater, 1995; Miller, 2001), and coalition signaling (Hagen and Bryant, 2003). Although the underlying cognitive and neural mechanisms that enable complex behavioral coordination are not fully understood (Fairhurst et al., 2010), social attention has been so far considered one of the most likely candidates (Macrae et al., 2008), together with action simulation (Gazzola and Keysers, 2009; Rizzolatti and Sinigaglia, 2010), and feelings of cooperation (Lieberman, 2007). Lim and Young (2006), using animal models, have proposed a three-step process to explain neurobiological mechanisms regulating the development of social relationships. First, the animal must be motivated to approach and engage another individual. Second, the animal must be able to identify the individual based on social cues through the formation of social memories; and third, with appropriate conditions, a bond can form, leading to preferential interaction with that individual. Thus, circumstances that promote interpersonal memory are necessary for social bonding.

We propose that dancing together in groups is one way to set the conditions for the first step in promoting human social bonding, namely, the motivation to approach an individual. The second component in promoting social relationships, the formation of social memories, has been demonstrated by Macrae et al. (2008), who showed that individuals had enhanced memory for person attributes as a result of engaging in synchronous movement. These researchers speculate that the mechanism that explains enhanced memory is greater attentional focus on the interaction partner who is moving in synchrony. Evidence supporting this notion has recently been found in an eye-tracking study in which participants observed people dancing either synchronously or asynchronously to a given musical beat (Woolhouse and Lai, 2014). In brief, Woolhouse and Lai (2014) found significantly increased gaze dwell times in cases where dancers’ actions synchronized to a regularly pulsed audio track, as opposed to instances in which dancers’ actions were asynchronous to the audio. The implications of these findings for the experiment reported here are addressed in the discussion to this paper.

Returning to the previous theme of the cognitive and neural mechanisms underpinning social bonding, recent research suggests that these processes are likely to be interconnected. Miles et al. (2010, p. 460) noted that synchrony or imitation probably leads to “…more interdependent, or ‘other-focused’ information processing”—an “attentional union”—which, in turn, enhances perceptions of intimacy. Therefore, the increased person perception found by Macrae et al. (2008) may represent one of several cognitive-neurological processes involved in social cognition. Likewise, the factors responsible for the heightened levels of compassion and altruistic behavior found by Valdesolo and DeSteno (2011) may represent a complementary process responsible for bonding together groups of individuals. However, although the social-bonding effects found in many studies of synchronized movement have been observed in different social contexts, the scientific literature on dance has yet fully to examine the mechanisms by which such bonding is actuated. By exploring the way in which group dancing creates similar effects to those identified in the synchronous- and rhythmic-movement literature, the present study was designed partly to fill this gap. Moreover, we sought to do this in a realistic, non-lab setting, which, as D’Ausilio et al. (2015) have noted, can have tangible benefits: music (and we would argue, also dance) is able to balance the requirements of ecological validity and experimental control when used to investigate human social interaction and cognition.

Specifically, we aimed to test whether dancers whose movements were temporally aligned had enhanced memory for various attributes of each other; simply put, whether people remember more about each other when they dance to the same music. In this respect, this paper develops the study of Macrae et al. (2008), which found that participants recalled more target words, and were more likely to recognize the experimenter, when the experimenter’s and participant’s hands waved in phase versus anti-phase, or did not wave at all. If a similar effect of improved recall for details of individuals with whom individuals danced in synchrony was also found, it would indicate that dance—along with music—is an interactive medium that exploits the effects of a more general capacity for coordinated movement. And, consequently, if enhanced memory between individuals can be demonstrated to arise from collective dancing, then it is possible that a motivation for dancing in groups is because it enhances memory for one another, thereby facilitating social bonding and cohesion.

Our study utilized the fact that, in general, music and dance in all world cultures has a periodic, rhythmic framework (Leman and Naveda, 2010; Naveda and Leman, 2010), a quality that facilitates interpersonal entrainment (Clayton et al., 2005). Furthermore, people who dance to the same piece of music are likely to have shared affective experiences (Koelsch, 2010), which, in turn, may enable them to attend to one another to a greater degree. As a result, we hypothesized that there would be greater memory for person attributes between those dancing to the same music than those dancing to different music. We examined the effects on memory for person-attributes in an experiment using “silent-disco” technology, which enabled us simultaneously to transmit two different songs, A and B (at different tempi), to two subgroups of dancers, one receiving Song A, the other Song B. The technology allowed us to conduct a real-world study with experimental rigor, and thus, arguably, to explore the effects of collective dancing on participants who were more likely to exhibit spontaneous “natural” behaviors than those taking part in purely lab-based studies.

## Dance Experiment

### Method

The silent-disco technology employed in the study consisted of a multichannel wireless transmitter unit and sets of wireless headphones capable of receiving a signal on more than one channel. Here, a simple two-channel set-up was used, the wireless transmitter having a range of greater than 100 m and each headphone set having a signal-to-noise ratio of greater than 75 dB. The experiment consisted of four sessions each with ten different participants, in which the effect of group dancing on memory for person attributes was tested. Using silent-disco headphones, in each session five participants danced to music at a faster tempo, while the remaining five participants danced to music at a slower tempo. A marked-up dance floor controlled the participants’ proximity to one another. This ensured that within the experiment sessions each participant was brought into close proximity with every other dancer, and allowed distance on the dance floor to be taken into account within the analysis. The memory targets used in the experiment were different colored sashes worn by the participants, half of which also prominently displayed large symbols of cats.

### Ethical Statement

Ethics approval for this study was granted by the Faculty of Music Ethics Committee, University of Cambridge, UK.

### Participants

The participants were non-professional dancers, and included 23 females and 17 males (mean age = 31.4; *SD* = 11.2; range = 18–60). The female/male distribution, mean age, and age SD per group within each trial was as follows. Trial 1: Group A, 3F/2M, mean age = 21.6, *SD* = 2.1; Group B, 1F/4M, mean age = 20.2, *SD* = 1.8. Trail 2: Group A, 4F/1M, mean age = 38.6, *SD* = 11.2; Group B, 4F/1M, mean age = 36.8, *SD* = 9.4. Trail 3: Group A, 3F/2M, mean age = 26.2; *SD* = 2.3; Group B, 2F/3M, mean age = 25.2; *SD* = 2.6. Trial 4: Group A, 3F/2M, mean age = 45.0; *SD* = 12.1; Group B, 3F/2M, mean age = 37.4, *SD* = 9.9. Expressed as percentages, where 100% = everyone knew everyone and 0% = no one knew anyone, known/unknown pairings per trial were as follows: Trial 1 = 35.6%; Trial 2 = 2.2%; Trial 3 = 51.1%; Trial = 26.7%. Half were recruited from a weekly amateur dance class held within the host university, while the remainder were recruited on a University Open Day, and included both students and members of the general public. The experiment was run in a spacious, artificially lit dance studio.

### Procedure

On entering the foyer of the dance studio, each participant’s face was photographed. Subsequently the participants were brought into the studio and randomly seated in a circle around the dance floor. Each was given silent-disco radio headphones set covertly either to Channel A or Channel B. The covert headphone setting meant that participants were unaware that they had been assigned to one of two groups, either A or B. There was then a short sound check during which headphone volumes were adjusted to comfortable levels.

**FIGURE 1:**
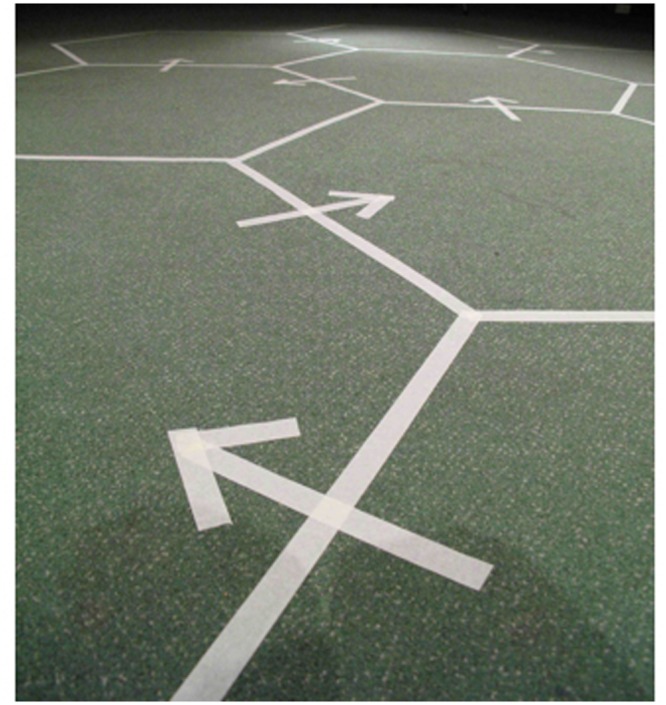
**Photograph of the dance floor showing the taped hexagons.** The hexagons were connected by a series of arrows outlining a path to be followed by the participants.

Prior to participants’ arrival, the dance floor had been divided into a series of 10 interlocking hexagons using white adhesive tape, each hexagon measuring 1.25 m in diameter. The hexagons were compactly configured, and a series of arrows delineated a path from one hexagon to another to be followed by participants during the experiment; see **Figure 1**. Before the experiment, participants were instructed to move between the hexagons via the arrows on the dance floor every time they heard a 2-s pause in the music. The 2-s pauses separated 10 periods of dancing, each 45 s in duration. The arrows were arranged so as to ensure that every participant was brought into a neighbor relationship with every other participant at least twice during the experiment. The marked-up dance floor and arrows enabled the distances between participants to be control, and, importantly, to equalize as far as was practicable the amount of time that each participant spent in close proximity with others dancing either to the same or different music. Participants were instructed to dance freely with their eyes open, interact with one another, and, if possible, relax and enjoy themselves. Lastly, so as to eliminate physical-interaction effects, participants were also requested to dance without making bodily contact.

**FIGURE 2:**
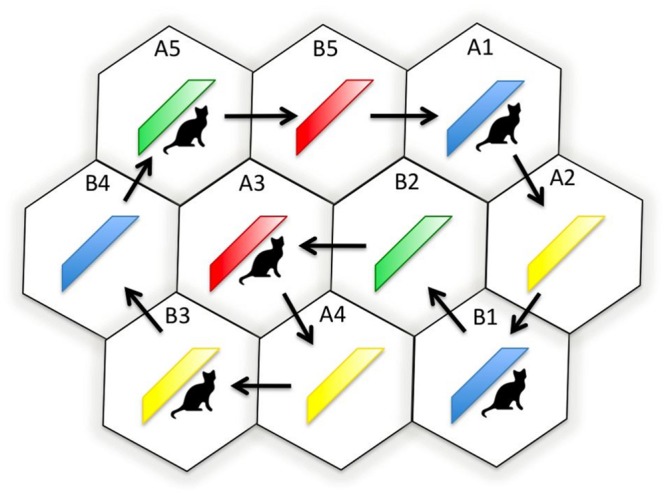
**Schematic diagram showing the dance floor hexagons, the initial positions, and group membership of the dancers (A1, A2, B1, B2, and so on.), the sash color worn by each dancer (blue, yellow, green, or red), and whether or not the dancer wore a cat symbol**.

Membership of either Group A or B depended on whether a participant’s headphones were set to Channel A or B, which, in turn, determined their starting position on the dance floor. After participants were positioned on the dance floor, the studio’s normal lighting was switched off and ultra violet lighting switched on. A red, yellow, blue, or green sash was then placed on each dancer in a pseudo-random order; see **Figure 2**. Ultra violet light was used during sash placement in order to restrict color observation to the dancing phase of the experiment (all sashes appeared gray under ultra violet light). Had normal lighting conditions been present during sash placement, it would not have been possible to ensure that observed memory effects had solely resulted from the dance phase of the experiment. As previously mentioned, half the sashes also carried rectangular cat symbols measuring 10 cm × 14 cm; see **Figure 2**.

**FIGURE 3:**
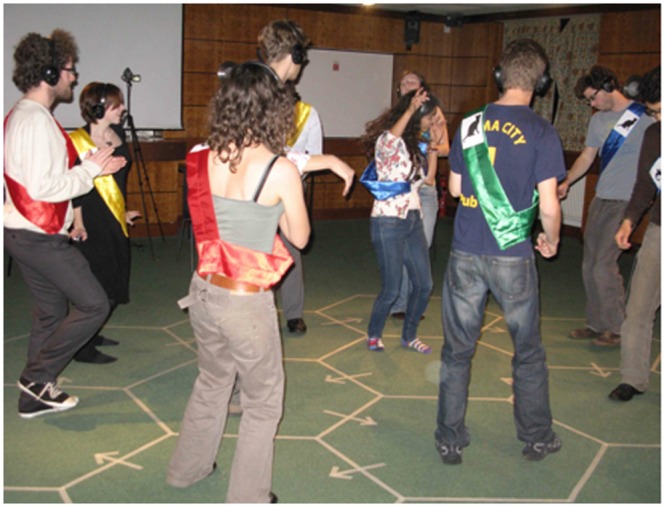
**Photograph of the participants taken during the experiment; note the silent-disco radio headphones, sashes, symbols, and marked up hexagonal dance floor used to control the proximity of the dancers**.

Normal studio lighting was switched back on as the music and dancing commenced; the studio lighting ensured that participants could see each other’s sash colors and cat symbols; see **Figure 3**. Music on Channel A was transmitted to Group A, while at the same time music on Channel B was transmitted to Group B. The two types of music were differentiated by artist, tempo, lyrics, and mood. Channel A music was *Wannabe* (1996) by British pop girl-band “Spice Girls”; music on Channel B was *Lady in Red* (1986) by Argentinian-born British singer-songwriter Chris de Burgh. The tempo of *Wannabe* was 121 bpm, the mood lively and upbeat. In contrast, the tempo of *Lady in Red* was 69 bpm, the mood subdued and sentimental. The two tempi were chosen because they lacked a simple integer ratio relationship, and therefore created asynchronous dancing between the two groups.

The experiment finished after each participant had occupied all 10 hexagons on the dance floor, i.e., after one complete cycle of the dance-floor pattern. The dancing lasted approximately 8 min (10 hexagon positions × 45 s = 7 min and 50 s). As soon as the music stopped for the final time, the studio lights were switched off, and participants’ sashes, symbols and headphones removed, again ensuring that the sash colors and symbols were only seen while the participants danced. Following the removal of the sashes, symbols and headphones, normal lighting was switched back on and the participants asked to return their seats. During the dancing, the participants’ seats were turned through 180°, forming an outward-facing circle.

During the 8-min dance phase of the experiment, questionnaires incorporating the colored photographs of the participants were prepared and printed. As soon as the participants were seated (looking away from one another), each was given a pencil and a copy of the questionnaire to complete. Participants were requested to recall the sash color and possible symbol associated with each dancer, identified by their photograph. The questionnaire also asked participants to indicate which dancers they knew prior to the experiment. The participants had 10 min in which to complete the memory task and questionnaire. In order to avoid participants adopting conscious memory strategies, only after completing the dancing were they made aware of the experiment’s memory component. Participants were debriefed and informed of the hypothesis of the experiment after completing the questionnaires.

## Results

A score of 1 was awarded for each correctly remembered sash color, and 1 for whether a participant wore a symbol; 0 was awarded for each incorrect response. Given that for each participant there were four same-music members and five different-music members, there was an increased probability that participants could have guessed the symbol and sash color of different-music members than same-music members (five different-music versus four same-music). Accordingly, each participant’s mean same- and different-music scores for symbol and sash color were expressed as percentages prior to analysis. Missing responses were modeled at chance: 0.5 for symbol (either symbol or no symbol), and 0.25 for sash color (one of four possible colors); missing responses accounted for 86 of the 720 possible responses, approximately 12%.

We investigated whether memory for sash color and symbol had been affected by dancing to the same piece of music in two separate analyses, one for sash color, the other for symbol. In addition, we took into account possible positive effects on memory of higher levels of arousal that may have resulted from dancing at the faster tempo (121 bpm versus 69 bpm; see Lambourne and Tomporowski, 2010). Accordingly, we conducted two full factorial repeated-measures ANOVA’s on participants’ scores (*n* = 40), with *Music* (same or different) as the within-group variable, and *Tempo* (fast or slow) as the between-group variable.

### Sash Color

There was a significant main effect of *Music* [*F*(1,39) = 4.229, *p* < 0.05, η^2^ = 0.048], but not of *Tempo* (*F* < 1). Participants who danced to the same music remembered each other’s sash colors to a greater degree than those who danced to different music; the tempo of the music did not have a significant effect on memory. With respect to sash color there was no interaction of *Music* with *Tempo* (*F* < 1).

### Sash Symbol

There was a significant main effect of *Music* [*F*(1,39) = 5.006, *p* < 0.05, η^2^ = 0.039], but not of *Tempo* (*F* < 1). As with sash color, participants who danced to the same music remembered each other’s sash symbols to a greater degree than those who danced to different music; the tempo of the music did not have a significant effect on memory. And similarly, as with sash color, for sash symbol there was no interaction of *Music* with *Tempo* (*F* < 1).

**FIGURE 4:**
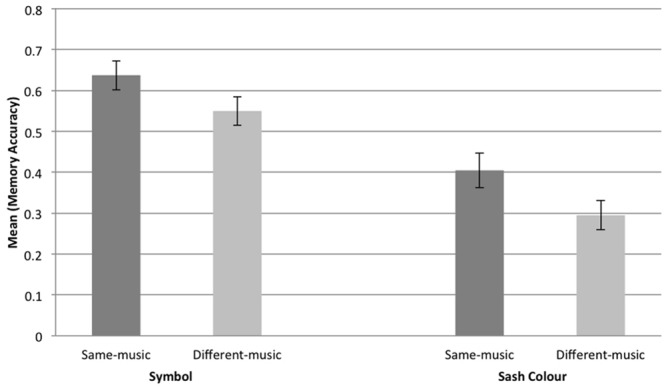
**Graph showing the mean response and standard error for symbol and sash color memory, same music, and different music**.

In sum, there was no effect of tempo on participants’ memory performances, whereas participants who danced to the same music, and therefore at the same tempo, exhibited greater inter-personal memory than those who danced to different music (and at a different tempo). Memory for symbol was greater than memory for sash color. This is perhaps not surprising given that the two targets used in the memory task, color and symbol, involved different memory loads: four colors of sash were present and all participants wore sashes; half the participants wore the cat symbol, half did not. Hence, overall performance on symbol was expected to be higher than performance on color, and, indeed, this was the case: the mean score for symbol was 0.59; for color 0.35. However, the memory advantage for same music over different music held for both color and symbol: the means for same-music symbol and color memory were, respectively, 0.64 and 0.40; the respective different-music means were 0.55 and 0.30. See **Figure 4**.

### Familiarity and Proximity

In order to explore whether prior familiarity and/or proximity on the dance floor had contributed to the results, we combined the memory performances of participants for sash color and symbol. With respect to prior familiarity, scores of participants who knew each other were then excluded and the analysis rerun; which is to say, participants were not excluded in the secondary analysis, simply scores where prior familiarity existed. For example, if Participant A had scores for Participants B, C, and D, and A knew B, A’s revised score would have been the average of C and D (i.e., only A’s score for B would have been excluded). Again, there was a significant main effect of the key factor *Music*: *F*_(1,39)_ = 7.894, *p* < 0.01. *Tempo* was not significant (*F* < 1).

To test whether the results had been influenced by proximity on the dance floor, the original scores were linearly weighted to reflect the amount of time each participant had spent one hexagon, two hexagons or three hexagons away from every other participant throughout the experiment. As before, there was a main effect of *Music* [*F*_(1,39)_ = 11.287, *p* < 0.005], but not of *Tempo* (*F* < 1), indicating that overall proximity had little noticeable effect. These and other findings reported above are now discussed.

## Discussion

The results of the experiment are consistent with the hypothesis that people who dance to the same piece of (in-tempo) music are more likely to recall various attributes of one another than those who dance to different (out-of-tempo) music. An apparent motivation, therefore, for people to engage in same-tempo group dancing may be to enhance person perception, and so provide the necessary conditions under which social bonding can occur. Given that participants danced freely, rather than in a synchronized manner, it is also possible that a general, albeit loose, coupling mechanism was responsible for the enhanced memory effect, rather than one relying upon precisely matched movements, as indicated in previous synchronization studies (e.g., Macrae et al., 2008). Moreover, as participants were encouraged to interact and observe each other on the dance floor, it seems likely that this coupling mechanism must relate to vision—participants did not converse or physically touch during the experiment, leaving vision as the only means by which pertinent information could be acquired.

Despite vision seemingly being key to our results, the experiment was not able to show whether same-music participants observed each other *more* than different-music participants. However, if we assume that a purpose of group dancing is to increase joint attention, it is reasonable to propose that participants’ gazes may have been predominantly directed towards other same-tempo dancers and away from different-tempo dancers. Indeed, results from a recent eye-tracking study exploring music-dance synchrony provide strong evidence that this is likely to have been the case.

Woolhouse and Lai (2014) investigated people’s eye-movements whilst observing pairs of laterally positioned dancers dancing synchronously or asynchronously to a musical beat, i.e., moving either in or out of tempo with the music the observer was hearing. Specifically, they tested two hypotheses: that enhanced memory for person attributes is the result of (1) increased gaze time between in-tempo dancers, and/or (2) greater attentional focus between in-tempo dancers. Woolhouse and Lai’s (2014) findings were consistent with the first hypothesis—music-dance synchrony resulted in significantly greater gaze times than music-dance asynchrony, and thus, they inferred, was likely to lead to increased memory for the attributes of those dancing together in time. In addition, they found a preference for upper-body fixations over lower-body fixations across both synchronous and asynchronous conditions. A subsequent, single-dancer eye-tracking study, also reported by Woolhouse and Lai (2014), investigated fixations across different body regions, including face, torso, legs, and feet. Significantly greater gaze times were recorded for face and torso than for legs and feet.

Recollect that in our silent-disco experiment, upon being presented with photographs of co-dancers’ faces, participants had to recall two memory targets, sash color and symbol, both of which were located on the upper body. In light of Woolhouse and Lai’s (2014) finding that dancers’ faces and torsos attracted greater gaze times than lower body regions, it is perhaps not surprising that participants’ same-tempo *and* different-tempo memory performances were above chance. In general, it appears to have been the case that the overall tendency for upper-body fixations led participants in our experiment to form mental associations between faces (subsequently presented in photographs) and memory targets, irrespective of synchrony. In addition, Woolhouse and Lai’s (2014) study also offers a plausible explanation for our finding that participants were more likely to recall the memory targets of those with whom they danced in time rather than out of time. As per Woolhouse and Lai’s (2014) experiment, in which music-dance synchrony resulted in significantly greater gaze times than asynchrony, our experiment resulted in participants exhibiting enhanced recall for those with whom they danced in time as opposed to out of time. The implication being that mutual gaze and dwell times between people who dance in time is significantly greater than between those who dance out of time. Of course, without data from mobile eye-tracking systems, which, given their physical presence around the eyes may inhibit dancing, this proposal is conjectural to some degree; however, the evidence for the existence of a vision mechanism linking gaze, human movement synchrony, and interpersonal memory would seem to be compelling.

Although, to our knowledge, this is the first recorded instance of enhanced interpersonal memory in the context of music and dance, our findings are consistent with previous studies involving recollection of person attributes, behavioral coordination and synchrony (e.g., Macrae et al., 2008). Moreover, our results with respect to familiarity and dancer proximity suggest that the proposed vision mechanism is relatively robust. Previously established relationships and/or friendships did not result in participants recalling memory targets disproportionately. Similarly, proximity on the dance floor appears not to have had a significant effect. This may have been due to the dance floor having good lines of sight, enabling non-adjacent dancers to see one another clearly; see **Figure 3**. Moreover, the manner in which participants were required to move around the dance floor (45 s per hexagon) brought them into close proximity, and thus effectively ‘mixed up’ the participants during the dance. Nor was there an effect of dancing at the faster tempo on memory target recall, which might have been anticipated based on research examining the correlation between exercise and cognitive performance (Lambourne and Tomporowski, 2010). In essence, if there was an effect of physiological arousal, it appears to have affected all participants equally, irrespective of the tempo at which they danced.

While the ability of individuals to entrain, and thus attend to one another, is likely to have been most strongly affected by the meter and tempo of the music, factors other than tempo may have contributed to our results—semantic/lyric differences, for example, could have led participants to express themselves with dissimilar dance gestures. However, eliminating semantic factors in music and musical entrainment, e.g., by using the same song at different tempi, may not be that trivial, since tempo is itself a component of musical meaning (Koelsch et al., 2004).

In the post-experiment phase, we obtained only informal information concerning whether participants knew that some people within the experiment were dancing at a different tempo, and therefore there is a limited amount that can be inferred from this feedback. However, many reported not realizing that some of their fellow dancers were dancing to different music—few participants had had previous experience of silent discos, and even fewer expected there to be multi-music and multi-tempi components to the experiment. In other words, most participants appear to have assumed that everyone was dancing to the same music, which, arguably, reinforces the notion that the memory effects we did observe were, to some extent, incidental, and not the result of explicit knowledge or conscious strategies adopted by all participants. Some participants did report finding the memory task difficult, which is perhaps understandable given that the dancing lasted only 8 min, and that they had no prior knowledge of the post-experiment memory task. Nevertheless, despite these difficulties, the results suggest that something as commonplace as dancing in time with other people significantly enhances memory for person attributes.

More generally, the results of our study support the conjecture that at least one significant, and possibly evolutionarily adaptive, function of music and dance is for bonding groups that extend beyond immediate family (Dissanayake, 2000; Nettl, 2005; Cross, 2011; Kaufman Shelemay, 2011). The ecologically grounded nature of our study, achieved by using a non-lab dance environment, and employing relatively large numbers of dancers within each trial, extends the scope of previous interpersonal entrainment research into a real-world setting.

### Summary

This paper demonstrates that dancing together in time can lead to increased recall of person-related memory targets, and therefore builds on the findings of previous studies showing that enhanced memory effects can arise in situations where movements are synchronous. Furthermore, our results suggest that the experience of group dancing is motivated by similar processes to those referred to in the literature on synchronized movement (and possibly also in communicative interaction in speech; see, Shockley et al., 2009). Dancing in groups is self-evidently a case where individuals synchronize and coordinate their movements in time, and, as noted at the outset of this paper, there is a substantial literature advancing the notion that a major role of collective dance is the affirmation and/or transformation of social bonds. However, to our knowledge it has not previously been proposed that group dancing should lead to (1) modifications in visual attention, and, as a result, (2) enhanced memory for attributes of those with whom we are dancing. Certainly it is the case that enhanced memory effects have been previously observed in synchronization tasks (e.g., Macrae et al., 2008), but not between individuals engaging in collective dance, an ecologically valid and relatively sophisticated cultural activity in comparison to laboratory studies. Should this finding be entirely unexpected, however? Arguably not: scholars, particularly those engaged in studying cultures other than contemporary urban Western societies, have for some time been aware that dance (and music) are more than mere pastimes. Rather, these activities underpin a range of individual and social functions, and are integral to the “healthy” functioning of the cultures they have explored. As the ethnomusicologist Thomas Turino (1999, p. 234) puts it,

The subtle rhythmic patterns—basic to how we speak, how we walk, how we dance, how we play music—are unspoken signs of who we are, whom we resemble, and thus whom we are with. Conversely, divergences in kinesic and other features of social style directly identify outsiders, those who are not like us… Sonic and kinesic iconicity, or lack thereof, however, comes to the fore in participatory musical and dance occasions because in such occasions these signs are the focal point of attention.

Silent-disco technology offers considerable benefits to those wishing to construct controlled social-interaction experiments in complex real-world environments. Given the purpose for which they were originally designed (to enable large numbers of people to dance together energetically in party-like settings), silent-disco headsets are relatively robust, and thus applicable to a range of experimental setups and designs. Further studies could incorporate motion-capture in order to investigate how dancing to the same or different music translates into synchronous action; a cross-cultural element might explore the extent to which our current findings are generalizable or specific. The degree to which the former is the case, i.e., generalizable, will, we anticipate, be a major target of future research within the rapidly expanding area of rhythmic entrainment research.

## Author Contributions

MW: Study design and execution, data interpretation and analysis, manuscript drafting. DT: Study design and execution, data interpretation and analysis. IC: Manuscript drafting, data interpretation and analysis.

## Conflict of Interest Statement

The authors declare that the research was conducted in the absence of any commercial or financial relationships that could be construed as a potential conflict of interest.

## Footnotes

1 For many world cultures, music and dance are not separable cultural categories; for example, Merriam (1964, p. 275) quotes Gbeho as stating that in the indigenous music of the Gold Coast “If we speak of a man being musical we mean that he understands all the dances, the drums and the songs.”

## References

[B1] Brown S. Martinez M. J. Parsons L. M. (2004). Passive music listening spontaneously engages limbic and paralimbic systems. *Neuroreport* 15 2033–2037. 10.1097/00001756-200409150-00008 15486477

[B2] Catchpole C. K. Slater P. J. B. (1995). *Bird Song: Biological Themes and Variations*. Cambridge: Cambridge University Press.

[B3] Cirelli L. K. Einarson K. M. Trainor L. J. (2014). Interpersonal synchrony increases prosocial behavior in infants. *Dev. Sci.* 17 1003–1011. 10.1111/desc.12193 25513669

[B4] Clayton M. Sager R. Will U. (2005). In time with the music: the concept of entrainment and its significance for ethnomusicology. *ESEM Counterpoint* 1 1–45.

[B5] Cross I. (2008). Musicality and the human capacity for culture. *Music. Sci.* 12 147–167. 10.1177/1029864908012001071

[B6] Cross I. (2011). “Music as a social and cognitive process,” in *Language and Music as Cognitive Systems* eds Rebuschat P. Rorhrmeier M. Hawkins J. A. Cross I. (Oxford: Oxford University Press) 313–328.

[B7] D’Ausilio A. Novembre G. Fadiga L. Keller P. E. (2015). What can music tell us about social interaction? *Trends Cogn. Sci.* 19 111–114. 10.1016/j.tics.2015.01.005 25641075

[B8] Dissanayake E. (2000). “Antecedents of the temporal arts in early mother-infant interactions,” in *The Origins of Music* eds Wallin N. Merker B. Brown S. (Cambridge, MA: MIT Press) 389–407.

[B9] Fairhurst M. T. Janata P. Repp B. H. Stelzer J. Keller P. E. (2010). “fMRI investigation of dynamic cooperativity: synchronised finger tapping with an adaptive virtual partner,” in *Proceedings of the 11th International Conference on Music Perception and Cognition (ICMPC 11)* eds Demorest S. M. Morrison S. J. Campbell P. S. (Seattle, WA: ICMPC) 168–172.

[B10] Freeman W. J. (2000). “A neurobiological role of music in social bonding,” in *The Origins of Music* eds Wallin N. Merker B. Brown S. (Cambridge, MA: MIT Press) 411–424.

[B11] Gazzola V. Keysers C. (2009). The observation and execution of actions share motor and somatosensory voxels in all tested subjects: single-subject analyses of unsmoothed fMRI data. *Cereb. Cortex* 19 1239–1255. 10.1093/cercor/bhn181 19020203 PMC2677653

[B12] Hagen E. H. Bryant G. A. (2003). Music and dance as a coalition signaling system. *Hum. Nat.* 14 21–51. 10.1007/s12110-003-1015-z 26189987

[B13] Hove M. J. Risen J. L. (2009). It’s all in the timing: interpersonal synchrony increases affiliation. *Soc. Cogn.* 27 949–961. 10.1521/soco.2009.27.6.949

[B14] Kaufman Shelemay K. (2011). Musical communities: rethinking the collective in music. *J. Am. Musicol. Soc.* 64 349–390. 10.1525/jams.2011.64.2.349

[B15] Kirschner S. Tomasello M. (2010). Joint music making promotes prosocial behavior in 4-year-old children. *Evol. Hum. Behav.* 31 354–364. 10.1016/j.evolhumbehav.2010.04.004

[B16] Koelsch S. (2010). Towards a neural basis of music-evoked emotions. *Trends Cogn. Sci.* 14 131–137. 10.1016/j.tics.2010.01.002 20153242

[B17] Koelsch S. Kasper E. Sammler D. Schulze K. Gunter T. Friederici A. D. (2004). Music, language and meaning: brain signatures of semantic processing. *Nat. Neurosci.* 7 302–307. 10.1038/nn1197 14983184

[B18] Lambourne K. Tomporowski P. (2010). The effect of exercise-induced arousal on cognitive task performance: a meta-regression analysis. *Brain Res.* 1341 12–24. 10.1016/j.brainres.2010.03.091 20381468

[B19] Large E. W. Jones M. R. (1999). The dynamics of attending: how people track time-varying events. *Psychol. Rev.* 106 119–159. 10.1037/0033-295X.106.1.119

[B20] Leman M. Naveda L. (2010). Basic gestures as spatiotemporal reference frames for repetitive dance/music patterns in samba and charleston. *Music Percept.* 28 71–91.

[B21] Lieberman M. D. (2007). Social cognitive neuroscience: a review of core processes. *Annu. Rev. Psychol.* 58 259–289. 10.1146/annurev.psych.58.110405.085654 17002553

[B22] Lim M. M. Young L. J. (2006). Neuropeptidergic regulation of affiliative behavior andsocial bonding in animal. *Horm. Behav.* 50 506–517. 10.1016/j.yhbeh.2006.06.028 16890230

[B23] Macrae C. N. Duffy O. K. Miles L. K. Lawrence J. (2008). A case of hand waving: action synchrony and person perception. *Cognition* 109 152–156. 10.1016/j.cognition.2008.07.007 18755450

[B24] Merker B. (2000). “Synchronous chorusing and human origins,” in *The Origins of Music* eds Wallin N. Merker B. Brown S. (Cambridge, MA: MIT Press) 315–328.

[B25] Merriam A. P. (1964). *The Anthropology of Music.* Chicago, IL: Northwestern University Press.

[B26] Miles L. K. Nind L. K. Henderson Z. Macrae C. N. (2010). Moving memories: behavioral synchrony and memory for self and others. *J. Exp. Soc. Psychol.* 46 457–460. 10.1016/j.jesp.2009.12.006

[B27] Miles L. K. Nind L. K. Macrae C. N. (2009). The rhythm of rapport: interpersonal synchrony and social perception. *J. Exp. Soc. Psychol.* 45 585–589. 10.1016/j.jesp.2009.02.002

[B28] Miller G. (2001). *The Mating Mind: How Sexual Choice Shaped the Evolution of Human Nature*. London: Vintage/Ebury.

[B29] Naveda L. Leman M. (2010). The spatiotemporal representation of dance and music gestures using topological gesture analysis (TGA). *Music Percept.* 28 93–111. 10.1525/mp.2010.28.1.93

[B30] Nettl B. (2005). *The Study of Ethnomusicology: Thirty-One Issues and Concepts* 2nd Edn. Chicago, IL: University of Illinois Press.

[B31] Phillips-Silver J. Keller P. (2012). Searching for roots of entrainment and joint action in early musical interactions. *Front. Hum. Neurosci.* 6:26 10.3389/fnhum.2012.00026 PMC3288575 22375113

[B32] Richardson D. C. Dale R. (2005). Looking to understand: the coupling between speakers’ and listeners’ eye movements and its relationship to discourse comprehension. *Cogn. Sci.* 29 1045–1060. 10.1207/s15516709cog0000_29 21702802

[B33] Rizzolatti G. Sinigaglia C. (2010). The functional role of the parieto-frontal mirror circuit: interpretations and misinterpretations. *Nat. Rev. Neurosci.* 11 264–274. 10.1038/nrn2805 20216547

[B34] Sebanz N. Bekkering H. Knoblich G. (2006). Joint action: bodies and minds moving together. *Trends Cogn. Sci.* 10 70–76. 10.1016/j.tics.2005.12.009 16406326

[B35] Shockley K. Richardson D. C. Dale R. (2009). Conversation and coordinative structures. *Top. Cogn. Sci.* 1 305–319. 10.1111/j.1756-8765.2009.01021.x 25164935

[B36] Shockley K. Santana M.-V. Fowler C. A. (2003). Mutual interpersonal postural constraints are involved in cooperative conversation. *J. Exp. Psychol. Hum. Percept. Perform.* 29 326–332. 12760618 10.1037/0096-1523.29.2.326

[B37] Turino T. (1999). Signs of imagination, identity, and experience: a peircian semiotic theory for music. *Ethnomusicology* 43 221–255. 10.2307/852734

[B38] Valdesolo P. DeSteno D. (2011). Synchrony and the social tuning of compassion. *Emotion* 11 262–266. 10.1037/a0021302 21500895

[B39] Wiltermuth S. S. Heath C. (2009). Synchrony and cooperation. *Psychol. Sci.* 20 1–5. 10.1111/j.1467-9280.2008.02253.x 19152536

[B40] Woolhouse M. H. Lai R. (2014). Traces across the body: influence of music-dance synchrony on the observation of dance. *Front. Hum. Neurosci.* 8:965 10.3389/fnhum.2014.00965 PMC4253660 25520641

